# Enhancing Midwifery Students’ Knowledge and Skills in Communication, Counselling, and Therapeutic Approaches Through an Elective Pilot Course: A Mixed-Methods Study

**DOI:** 10.3390/healthcare13101180

**Published:** 2025-05-19

**Authors:** Metka Skubic, Tita Stanek Zidarič, Anita Jug Došler, Lucija Šerjak

**Affiliations:** Faculty of Health Sciences, University of Ljubljana, 1000 Ljubljana, Slovenia; metka.skubic@zf.uni-lj.si (M.S.); tita.zidaric@zf.uni-lj.si (T.S.Z.);

**Keywords:** elective pilot course, education, communication, counseling, therapeutic skills, e-learning, midwifery

## Abstract

**Background/Objectives**: Midwives are crucial in addressing complex women’s health issues, such as infertility, breastfeeding challenges, and neonatal health. An elective pilot course, “*Educational, Counseling, and Therapeutic Approaches in Midwifery*”, was designed to enhance midwifery students’ knowledge and skills in communication, counseling, and therapeutic skills via e-learning approaches. **Methods**: A mixed-methods approach was employed, combining pre- and post-testing to assess students’ skill development. In addition, guided reflective discussions were based on video and audio recordings of pre-prepared role-playing scenarios. Students worked in pairs, alternating roles as midwives and patients, to engage in real-life situations. During the reflective discussions, students critically analyzed their experiences of the consultation process, identifying their strengths and weaknesses, and reflecting on what went well and what could be improved in future interactions. **Results**: The initial findings revealed that students were overconfident in their skills, but through role-playing and reflective discussions, they recognized gaps in their knowledge and developed a deeper understanding of essential competencies. **Conclusions**: The elective pilot course proved effective in enhancing students’ knowledge and skills as counselors. These results emphasize the importance of integrating structured e-learning and educational strategies into midwifery training to improve care and health outcomes.

## 1. Introduction

The International Confederation of Midwives [1] defines a comprehensive range of competencies essential for all midwives, outlining the scope of midwifery practice and serving as the foundation for formal midwifery education. These competencies encompass various aspects of effective communication, education, and counseling, which are integral to providing midwifery care for patients, couples, and families.

### 1.1. Communication and Counseling

Counseling is a supportive process that provides a non-judgmental, understanding environment, helping individuals gain insight into their concerns and themselves. It differs from advice-giving, sympathy, or practical assistance by empowering clients to rely on their own resources for resolution [2]. In midwifery, counseling constitutes a crucial component of comprehensive care, addressing both informational and emotional needs. It encompasses a broad range of issues, requiring midwives to possess extensive counseling skills, including active listening, reflection, and redirection to facilitate effective communication [3,4].

Effective communication is essential in midwifery, particularly in the context of counseling, and requires knowledge, cultural sensitivity, and the use of appropriate language [4]. The most effective communication style is a patient-centered approach [5], where midwives see their role as supporting women in making informed choices by providing relevant information while fostering a meaningful, constructive relationship. Midwives who adopt this approach demonstrate empathy, authenticity, and non-judgmental attitudes toward patients, treating patients as equals. They are supportive, encouraging, and affirming, creating an environment where patients feel safe and comfortable.

### 1.2. Therapeutic Approaches

For some time, psychological and personal growth knowledge has been recognized as a valuable therapeutic tool in midwifery, with communication and counseling becoming integral to practice. As midwifery seeks greater autonomy, incorporating psychotherapeutic approaches and counseling skills could enhance emotional support and client care [6]. Midwives can assist women in exploring the sources of problems, addressing the impact of negative past experiences, providing information on available options, and supporting the development of strategies for a positive experience [7].

The midwife–patient relationship is central to midwifery care [8], as it fosters trust and security, and provides interpersonal support [9]. Availability, responsiveness, and synchronization are key factors in forming an emotional connection, which serves as an indicator of high-quality midwifery care [10]. Establishing rapport is particularly important in the early stages of a therapeutic relationship, as it helps create a trusting and safe environment. Effective therapeutic approaches utilize both verbal and non-verbal communication, using skills like active listening, providing clear information, attentiveness, empathy, respect, and the use of therapeutic touch [2,9]. Ultimately, the goal is to establish a collaborative partnership in which the patient feels heard and supported [2].

Such care is usually called emotional care, which is multifaceted, involving the assessment of emotional needs, emotional support, and therapeutic emotional labor, all of which are essential for promoting confidence and comfort [8]. It is crucial for midwives to listen to and support women in expressing their feelings about their experiences, as this can help address emotions such as fear, loss, and self-blame. Midwives should assist women in understanding the events, clarify any misunderstandings, and provide emotional validation to foster healing. Additionally, supporting women in exploring potential solutions, reinforcing positive coping strategies, and enhancing social support networks can help restore self-confidence and aid recovery after a traumatic experience [9,11]. Interventions like these highlight the midwife’s role in providing emotional care and are already incorporated into midwifery practice to some extent, making them easy to integrate into existing services without requiring extensive training [11].

### 1.3. Learning Approaches for Skills Teaching

Many authors emphasize that midwifery students and practicing midwives should receive formal education on how to successfully interact with patients [4,11], as midwives are ideally positioned to offer comprehensive maternity care, which includes the identification, management, and support of patients experiencing perinatal emotional distress [12]. However, while midwives express a willingness to assist and recognize the importance of providing emotional care, their ability to do so is often hindered by perceived deficiencies in competence, rather than a lack of interest [12]. There is a clear need to incorporate these so-called “soft skills”, such as effective communication, counseling, and therapeutic approaches, into midwifery curricula and courses to enhance professional development [4].

Soft skills, also known as “Core Competencies”, are behavioral and socio-emotional abilities such as creativity, problem-solving, communication, empathy, critical thinking, confidence, situational awareness, and teamwork. These skills are essential across various professions, including midwifery [13,14]. They can be developed through interactive and creative learning techniques that promote respectful maternity care, including discussion, brainstorming, demonstration, case studies, storytelling, personal experiences, videos, and role play [15,16], with role play being particularly effective in enhancing communication skills and empathy [17,18]. Role plays simulating real-life scenarios, similar to those encountered in clinical placements, are seen by students as valuable for future work, and engaging, as they offer a realistic learning opportunity in a safe and controlled environment [15,19]. After using interactive teaching methods, including simulation, debriefing is recognized as a crucial step that requires sufficient time to effectively conclude the process [20]. During debriefing, students can engage in self-analysis and receive constructive feedback from teachers and peers, which raises awareness of their own skills, encourages improvement, and provides information for practical application [16]. Despite the recognized importance of soft skills, challenges remain in their precise definition, assessment, and integration into curricula [13].

### 1.4. Study Aim

This study aimed to evaluate the implementation of the elective pilot course “Educational, Counseling, and Therapeutic Approaches in Midwifery” (ECTAM), developed within the University of Ljubljana’s “UL for a Sustainable Society—ULTRA” project, at the Department of Midwifery, University of Ljubljana, Slovenia. The focus of the study was on students’ experiences as midwifery counselors. It examined students’ perceived knowledge and skills in communication, counseling, therapeutic approaches, evidence-based practice, and practical midwifery skills before and after the course. The following research questions guided the study:

- What is the students’ perspective on the understanding and application of communication, counseling, and therapeutic approaches in midwifery?
- How do students assess their knowledge and skills in the areas of communication, counseling, and therapeutic approaches before and after completing the elective pilot course “Educational, counseling, and therapeutic approaches in midwifery”?

## 2. Materials and Methods

### 2.1. Study Design and Procedure

The study used a mixed-methods research design [21] combining qualitative and quantitative approaches to gain a comprehensive understanding of the pilot’s implementation and validate its findings. This approach is increasingly utilized in health sciences for socially complex interventions, particularly in health promotion, disease prevention, and bereavement counseling [22,23,24].

As part of our project, we selected both qualitative data (questionnaires with open-ended questions, student reflections, and notes) and quantitative data (questionnaires to measure impact before and after the course). The decision to employ a combined research approach, following the mixed-methods research methodology, was made to enhance the validity and reliability of the research findings. The results of both parts are presented in the Results section.

### 2.2. Structure and Implementation of the ECTAM Course

The elective pilot course was structured to support the integration of educational, counseling, and therapeutic approaches into midwifery practice. It integrates lifelong project-based e-learning through a blend of lectures, seminars, and practical exercises that emphasize experiential, ICT-supported, and interactive pedagogical methods to enhance student engagement and skill development.

Project-based e-learning is a digital instructional method where students engage in extended inquiry driven by real-world questions. It emphasizes independent and collaborative learning through two core elements: (a) students develop and explore their own research questions, and (b) they create digital products in response. The approach integrates online tools for communication, research, collaboration, and presentation [25,26].

The ECTAM course theoretical lectures introduced core concepts including education, communication, counseling strategies, therapeutic techniques, evidence-based practice, and the principles of project-based e-learning, allowing students to refresh and deepen their knowledge. In the seminars, students engaged in project-based e-learning and, under the guidance of teachers, developed clinical scenarios, which included different issues involving infertility, breastfeeding problems, and newborn examination. These scenarios were later used for role-playing counseling simulations during practical exercises, where students worked in pairs, one taking on the role of a midwife counselor and the other the role of a service user. The simulations also included necessary mannequins, such as newborns and pre-term babies, to increase realism.

Simulation-based learning, supported by digital materials (video and audio recordings of the role-play counseling simulations) and recording tools (Apple iPad devices), allowed students to record and analyze their counseling sessions. After each simulation, students provided peer feedback through an online evaluation form, followed by a debriefing session and self-reflection on their counseling process. Teachers also facilitated a guided debriefing discussion, offering constructive feedback and recommendations for improvement. Finally, each student completed the self-reflection form on the implementation of the counseling process.

### 2.3. Setting and Sample

The study included third-year midwifery students enrolled in the ECTAM course at the University of Ljubljana, Faculty of Health Sciences. Students were recruited through purposive sampling via an announcement during lectures, where details were provided about the study’s purpose, voluntary participation, and confidentiality. Interested students signed an informed consent form before data collection began.

A total of 30 students were invited to participate in the study and began the pre-course questionnaire. However, in accordance with ethical research principles and the voluntary nature of participation, 5 students exercised their right to withdraw at any time—a decision that was fully respected by the researchers. Consequently, the post-course questionnaire was completed by 25 students. The inclusion criteria for participation were enrollment as a third-year midwifery student, participation in the ECTAM course “Educational, Counseling, and Therapeutic Approaches in Midwifery”, and willingness to participate in the study.

### 2.4. Ethical Considerations

This study was conducted in accordance with the ethical principles outlined in the Declaration of Helsinki [27] and the Code of Ethics for Researchers at the University of Ljubljana, ensuring respect for human dignity, voluntary participation, and confidentiality of the collected data. Ethical approval was obtained from the institutional review board of the Midwifery Chair Board, Faculty of Health Sciences. Students were informed about the study’s purpose and procedures, and written informed consent was obtained. Before the ECTAM course, students signed consent forms for documenting the educational process (video recording) and participation in the study. The video material is stored with restricted access and is not used for commercial purposes.

### 2.5. Data Collection and Analysis

Data were collected between March and April 2024, following prior ethical approval. The study’s first part used a quantitative approach with two questionnaires: (1) pre-course and (2) post-course. These questionnaires were designed to assess students’ self-perceived knowledge and skills related to the topics covered in the ECTAM course. Each questionnaire included 6 items, as shown in Table 1, and used a 5-point Likert scale ranging from 1 (“strongly disagree”) to 5 (“strongly agree”). The questionnaires were developed specifically for this pilot course and, while not previously validated, were intended to track perceived changes in knowledge and skills before and after participation. The second part employed a qualitative approach with two instruments: (1) an assessment of students’ understanding of key concepts in education, counseling, and therapeutic approaches, which included questions like “What do you understand by the term “consulting approaches in midwifery” and where will you need knowledge and skills to perform consulting activities in your work in the field of midwifery?” and (2) a self-reflection tool for evaluating the counseling process. The research team, consisting of four teachers involved in the ECTAM course, distributed the questionnaires, explained the study’s purpose, and collected completed instruments. Content validity was verified through a literature review and pilot testing, where the questionnaires were found to be clear and understandable. Reliability and sensitivity were ensured with precise instructions, while objectivity was maintained through consistent criteria for rating scales, responses to closed-ended question types, and uniform, unambiguous instructions for completion.

For the quantitative data, the descriptive statistics (frequencies and percentages) were calculated, and the differences before and after the ECTAM course, as seen from the midwifery students’ perspective, were analyzed using the Kullbackov 2Î test, with statistical significance set at the 5% level. IBM SPSS Statistics 29.0.0.0 software (SPSS Inc., Chicago, IL, USA) was used for the analysis. For the qualitative data, thematic content analysis was conducted on open-ended questions and student reflections using MAXQDA 24.8.0 software (VERBI Software GmbH, Berlin, Germany). The analysis followed the six steps outlined by Braun and Clarke [28]. First, the researchers became familiar with the data through repeated reading. Next, initial codes were created to highlight important features across the dataset. These codes were then grouped into potential themes. The themes were reviewed to ensure they clearly represented the data and were different from each other. After that, the themes were defined and given names that described their main ideas. Finally, the analysis was written up by linking the themes to the research questions and course outcomes. This step-by-step process helped produce meaningful contextual insights into the course’s practical application.

## 3. Results

We present the results of our mixed-method research as an analysis of differences before and after the ECTAM course implementation, along with a self-assessment of the course execution by the students. The self-assessment was intended to enhance the statistical data by providing additional insights into the students’ perspectives on the course execution and its impact. The results of the statistical data analysis are shown in Table 1.

### 3.1. Knowledge and Skills in Professional Communication and Counseling Within Midwifery Practice

Since the course aimed to enhance students’ knowledge and skills in professional communication and counseling within midwifery practice, we wanted to gain insight into students’ views on counseling. Qualitative data collected from the students revealed that they conceptualized counseling in midwifery practice as a multifaceted process. Three primary types of counseling were identified: preventive counseling (focused on health education such as family planning, contraception, sexuality, and nutrition), counseling for different life stages (including pregnancy, the postpartum period, and parenthood), and counseling related to medical interventions. Counseling was often associated with interactions between midwives and patients, particularly mothers, fathers, parents, and families, in settings such as gynecological practices, parenting schools, delivery rooms, and postpartum wards. Notably, students also emphasized the importance of counseling directed toward colleagues and co-workers, highlighting the collaborative nature of midwifery practice. In terms of counseling techniques, students described a process that involved providing information to support individuals in making informed decisions and navigating challenging situations, particularly related to pregnancy, labor, postpartum care, sexuality, and infant care.

Our quantitative findings further reinforced these insights by demonstrating a clear improvement in students’ self-assessment of their communication and counseling knowledge and skills after completing the course, which covered some of the areas they identified as important. As seen in Table 1 (Statement 1), there was a positive change in the students’ self-assessment of their professional communication skills in midwifery after the course. While the majority (56.7%) initially agreed that they lacked sufficient knowledge and skills, this number decreased significantly after the course: 64% disagreed with the statement. This improvement is statistically significant (Kullback 2Î test, χ^2^(3) = 20.672, *p* = 0.001), indicating that the course effectively improved students’ perceived knowledge and skills in this area. Similarly, the students’ perceived knowledge and skills in counseling showed substantial improvement (Table 1, Statement 2). Before the course, 42.9% of students felt confident about their knowledge and skills in this area. This number almost doubled to 84% after the course. This positive change is statistically significant (Kullback 2Î test, χ^2^(3) = 16.215, *p* = 0.001).

### 3.2. Knowledge and Skills in Therapeutic Approaches and the Delivery of Live Counseling Sessions

Before the course, students shared their insights on therapeutic approaches, highlighting key themes that shaped their understanding of this important aspect of midwifery. Psychological support was frequently emphasized, with students recognizing its role in addressing emotional needs, fostering empathy, and providing encouragement. Physical components, such as therapeutic touch, were also mentioned, along with non-verbal cues, including eye contact, accepting body posture, and assertive communication paired with active listening. These elements were seen as essential in fostering effective therapeutic relationships. Students further identified situations where therapeutic approaches were particularly beneficial, such as during complications in pregnancy or delivery, challenges with newborns, stillbirths, and infertility. They acknowledged that these approaches were invaluable for managing distress, pain, and emotional challenges, especially in women needing support during difficult moments.

Additionally, students recognized the importance of therapeutic approaches in maternity hospitals, delivery rooms, postpartum wards, and specialized units like neonatal intensive care units and those for pathological pregnancies, where a holistic approach was deemed especially crucial.

The recognition of these therapeutic components laid the groundwork for understanding students’ perceived knowledge and skills in this area. Following the course, significant improvements in students’ self-assessment of their therapeutic skills were observed. As shown in Table 1 (Statement 3), students’ perceived knowledge and skills in therapeutic approaches to midwifery improved substantially after the course. Prior to the course, 60% of students felt they lacked sufficient knowledge and skills in this domain. However, this number decreased dramatically to just 16% after the course, marking a statistically significant change (Kullback 2Î test, χ^2^(3) = 15.370, *p* = 0.002). This indicates that the course was effective in enhancing students’ understanding and capabilities in therapeutic approaches. Similarly, students’ confidence in their ability to perform live therapeutic counseling sessions also improved significantly. Before the course, 44.8% of students strongly agreed that they wanted to improve their counseling skills, and 41.4% agreed. After the course, the percentage of students who strongly agreed dropped to 28%, while the number of students who agreed increased to 72%. Notably, no students disagreed after the course, reflecting a positive shift in their self-perception of their developmental needs. This change was statistically significant (Kullback 2Î test, χ^2^(3) = 8.285, *p* = 0.04), as shown in Table 1 (Statement 4), further emphasizing the impact of the course on enhancing students’ abilities and confidence in live therapeutic counseling.

After participating in live therapeutic counseling sessions through role play, students identified areas for improvement, including delivering clear and appropriate communication tailored to the individual’s level of understanding, and enhancing therapeutic skills such as active listening, paraphrasing, and asking open-ended questions.

### 3.3. Evidence-Based Midwifery Practices in Educational, Counseling, and Therapeutic Approaches

Prior to the course, students perceived evidence-based practice as particularly important, especially in terms of the research required to implement effective midwifery practice. Its significance was discussed primarily in relation to staying updated with new knowledge across various areas of midwifery work, including counseling and therapeutic approaches.

The course had a significant positive impact on students’ familiarity with evidence-based midwifery in the areas of educational, counseling, and therapeutic approaches (Table 1, Statement 5). The proportion of students who stated that they were familiar with the topic increased from 41.4% before the course to 68% after the course. This change is statistically significant (Kullback 2Î test, χ^2^(4) = 12.303, *p* = 0.015).

Due to the themes included in the course content (breastfeeding, infertility, and systematic newborn examination), students reported having either basic or advanced knowledge, which allowed them to apply evidence-based practices and information during the therapeutic counseling sessions. However, they identified a need for more information on infertility, particularly regarding treatments and protocols used in Slovenia. They recognized the importance of evidence-based practice, particularly during the debriefing and peer-to-peer evaluations, when errors such as providing incorrect or flawed information, using inappropriate approaches, or lacking effective communication skills were highlighted.

### 3.4. Knowledge and Experience with the Project-Based E-Learning

The course significantly improved students’ understanding of project-based e-learning (Table 1, Statement 6). The percentage of students who were familiar with the method increased dramatically from 43.3% before the course to 96% after. This improvement is statistically significant (Kullback 2Î test, χ^2^(4) = 31.084, *p* = 0.001).

During the project-based e-learning, students identified knowledge gaps they had not previously recognized. These gaps were particularly evident in scenarios that presented more complex and less common situations in midwifery practice. As these scenarios extended beyond the typical scope of everyday practice, students sometimes found it challenging to provide accurate information to service users. Consequently, many students expressed feelings of incompetence, acknowledging that their knowledge in these more specialized areas was insufficient.

In the self-evaluation process, students noted that the most discomforting aspect of the role-play simulation was the recording of their interactions, as this was not a standard component of their usual laboratory exercises. However, upon reviewing the recordings and recognizing mistakes made during the counseling process, many students valued the opportunity for reflection. Several students even suggested that incorporating recorded simulations into the curriculum for all laboratory exercises would be beneficial, emphasizing the value of self-reflection and the potential for enhanced learning through this approach. They also appreciated the safe environment among peers, where they felt comfortable, and recognized the value of the mentorship provided by the teachers present.

## 4. Discussion

The mixed-methods approach provided a comprehensive view of the impact of the ECTAM course. Our assessment was designed to determine whether the course met its primary objective—specifically, whether students had developed the necessary knowledge and skills for future professional practice in counseling and therapeutic approaches within midwifery. It was equally important to understand how students perceive these approaches, as their perceptions shape their self-assessment of their knowledge and skills. The results suggest that students have a strong understanding of these concepts and recognize the importance of soft skills in their midwifery practice.

Our findings strongly indicate that the ECTAM course had a positive impact on students’ perceived knowledge and skills in several key areas. All reported improvements were statistically significant (*p* < 0.05), reducing the likelihood that the changes occurred by chance. The *p*-values were generally very low (often *p* < 0.005 or even *p* < 0.001), suggesting strong evidence of a real effect. Overall, the data support the conclusion that the course was effective in achieving its intended learning outcomes, enhancing students’ perceived competence across a range of essential midwifery skills and knowledge areas.

These findings align with previous studies suggesting that learning and applying soft skills positively affect midwives’ self-confidence, self-awareness, and competence [18], better preparing students for future employment and success [19], particularly in managing emotionally challenging interactions with patients [17]. Role-play, in particular, improves students’ communication and counseling skills, serving as a valuable tool by providing various settings and scenarios for practice [18,29]. In our study, students generally expressed high satisfaction with the project-based e-learning, including role-play. They found the experience both interesting and valuable. The experience of being “in charge of the situation” and having to use knowledge, skills, and competencies to ensure quality midwifery care in a safe and controlled environment was something that was important to them, as also noted in some other studies [30,31]. Some students suggested incorporating actors or real users of midwifery services to enhance the realism of the simulations, an idea also supported by other studies [15,19].

Students frequently highlighted the importance of counseling across different life stages and health conditions, with a particular focus on practical techniques and the overall approach to counseling. They identified a range of therapeutic approaches in midwifery, including psychological and physical support, communication strategies, and the management of complications and special circumstances during pregnancy, childbirth, and the postpartum period. Therapeutic counseling in midwifery requires delivering information clearly and comprehensively while engaging with women’s personal experiences, values, and beliefs to support informed decision-making and assist them in achieving their health goals [3,4]. This process is complex and demands skilled professionals. Some students in our study reported challenges during simulated counseling sessions, noting uncertainty about how the session would evolve. They identified knowledge gaps they were previously unaware of, especially when encountering unfamiliar situations. Additionally, they proposed specific themes for future counseling simulations, such as stillbirths, miscarriages, and sexual health issues.

The thematic analysis revealed that counseling and therapeutic approaches in midwifery are widely integrated into practice, encompassing various forms, target groups, and content. This practice is essential in daily healthcare settings, where therapeutic and counseling processes are offered to patients at different life stages. As specialized psychological and psychiatric services may not always be accessible to all women, psychoeducational and other therapeutic approaches within midwives’ competencies provide a practical and cost-effective solution [7].

These findings underscore the importance of integrating communication, counseling, and therapeutic approaches into midwifery curricula to better prepare students for real-world practice.

### Study Limitations

Although the learning experience was generally positive, several limitations were encountered during the preparation and implementation of the educational program. One such limitation was students’ unfamiliarity with the working methods used, which led to reluctance in engaging with activities such as recording, role-playing, and scenario preparation. Furthermore, a lack of prior preparation and practice in a simulated environment contributed to student distress when they encountered real clinical situations. It is important to clarify that all activities took place in a simulated learning environment and not in actual clinical settings; therefore, the study did not examine students’ distress in real clinical situations. This remains an open question and could be explored in future research, particularly in relation to how simulated experiences prepare students for real-world clinical practice. Opportunities for improvement include enhancing individual and paired work, as well as promoting discussions on technical challenges, pedagogical approaches, and student experiences throughout the learning process. Additionally, it is important to note that the sample size slightly decreased after the course, from 30 students to 25. While this reduction does not invalidate the results, it should be considered as a factor. Finally, the relatively low number of participants overall must also be taken into account when interpreting the findings.

Another limitation is the reliance on students’ self-assessment of their knowledge and skills, without an external or objective evaluation. Including such assessments in future research could provide a more robust measure of learning outcomes. Additionally, comparing self-assessments with external evaluations using the same Likert scale could offer insights into the accuracy of students’ perceived progress. As all midwifery students in Slovenia are enrolled in our program, the participant pool reflects the entire national student population; however, future studies could consider involving international students to broaden the applicability of findings. Potential response bias should also be acknowledged, as students may have felt some pressure despite the anonymity of the questionnaires.

## 5. Conclusions

Based on the results of the mixed-method study assessing students’ perceived knowledge and skills in communication, counseling, and therapeutic approaches in midwifery practice, the findings indicate that the ECTAM elective pilot course was effectively planned, developed, and implemented, resulting in a significant enhancement in students’ knowledge and skills following the course. Despite some limitations, the study provided valuable insights into the benefits of integrating structured e-learning and simulation-based strategies into midwifery curricula. Such approaches have the potential to improve students’ soft skills, thereby contributing to the overall improvement of women’s healthcare in real clinical settings. The students’ feedback highlights the importance of incorporating complex, realistic scenarios into educational environments. Although these scenarios may initially induce discomfort or uncertainty, they ultimately present valuable learning opportunities. These experiences not only identify areas for improvement but also encourage students to deepen their understanding of less familiar, yet crucial, aspects of their professional practice.

## Figures and Tables

**Table 1:** Students’ assessment of their knowledge and skills before and after ECTAM course.

Statement in Pre/Post-Course Questionnaire	Pre-/Post-Course	I Don’t Agree at All (n, %)	I Don’t Agree (n, %)	I Can’t Decide (n, %)	I Agree (n, %)	I Completely Agree (n, %)	Total (n, %)	Kullbackov 2Î Test	*p*
1. I assess that I have insufficient knowledge and skills in the field of professional communication in midwifery.	Pre-course	0	5	5	17	3	30	20.672	0.001
		0%	16.7%	16.7%	56.7%	10%	100%		
	Post-course	0	16	6	3	3	25		
		0%	64%	24%	12%	5.5%	100%		
2. I consider that I have knowledge and skills in the field of midwifery counseling.	Pre-course	0	7	8	12	1	28	16.215	0.001
		0%	25%	28.6%	42.9%	3.6%	100%		
	Post-course	0	0	2	21	2	25		
		0%	0%	8%	84%	8%	100%		
3. I estimate that I have insufficient knowledge and skills in the field of therapeutic approaches.	Pre-course	0	3	7	18	2	30	15.370	0.002
		0%	10%	23.3%	60%	6.7%	100%		
	Post-course	0	12	8	4	1	25		
		0%	48%	32%	16%	4%	100%		
4. I would like to improve my knowledge and skills in the field of performing live counseling.	Pre-course	1	0	3	12	13	29	8.285	0.04
		3.4%	0%	10.3%	41.4%	44.8%	100%		
	Post-course	0	0	0	18	7	25		
		0%	0%	0%	72%	28%	100%		
5. I am familiar with evidence-based midwifery in the areas of educational, counseling and therapeutic approaches in midwifery.	Pre-course	1	5	11	12	0	29	12.303	0.015
		3.4%	17.2%	37.9%	41.4%	0%	100%		
	Post-course	0	2	3	17	3	25		
		0%	8%	12%	68%	12%	100%		
6. I know the project-based e-learning method.	Pre-course	7	13	6	4	0	30	31.084	0.001
		23.3%	43.3%	20%	13.3%	0%	100%		
	Post-course	1	1	3	16	4	25		
		4%	4%	12%	64%	16%	100%		

Legend: n—number; %—percentage; *p*—statistical significance.

## Data Availability

The data presented in this study are available on request from the corresponding author and are not publicly available to maintain participants’ anonymity.

## Abbreviation

The following abbreviations are used in this manuscript:

ECTAM	Educational, Counseling, and Therapeutic Approaches in Midwifery
